# Rank distributions: Frequency vs. magnitude

**DOI:** 10.1371/journal.pone.0186015

**Published:** 2017-10-05

**Authors:** Carlos Velarde, Alberto Robledo

**Affiliations:** 1 Instituto de Investigaciones en Matemáticas Aplicadas y en Sistemas, Universidad Nacional Autónoma de México, Mexico City, Mexico; 2 Instituto de Física y Centro de Ciencias de la Complejidad, Universidad Nacional Autónoma de México, Mexico City, Mexico; Universidad Rey Juan Carlos, SPAIN

## Abstract

We examine the relationship between two different types of ranked data, frequencies and magnitudes. We consider data that can be sorted out either way, through numbers of occurrences or size of the measures, as it is the case, say, of moon craters, earthquakes, billionaires, etc. We indicate that these two types of distributions are functional inverses of each other, and specify this link, first in terms of the assumed parent probability distribution that generates the data samples, and then in terms of an analog (deterministic) nonlinear iterated map that reproduces them. For the particular case of hyperbolic decay with rank the distributions are identical, that is, the classical Zipf plot, a pure power law. But their difference is largest when one displays logarithmic decay and its counterpart shows the inverse exponential decay, as it is the case of Benford law, or viceversa. For all intermediate decay rates generic differences appear not only between the power-law exponents for the midway rank decline but also for small and large rank. We extend the theoretical framework to include thermodynamic and statistical-mechanical concepts, such as entropies and configuration.

## Introduction

Ranking data that originates from apparently disconnected subjects in many fields —astrophysical, geophysical, ecological, biological, technological, financial, urban, social, etc.— has revealed universal patterns [1, 2] and opened intriguing questions about their origin. The empirical law of Zipf [3, 4] for the numbers of occurrence (frequencies if normalized) of words in texts has played a central role in the development of this widespread research topic of multidisciplinary complex systems. Zipf’s law has been found to be (approximately) followed by many sets of ranked data outside linguistics, that record the number of occurrences [5] of other types of items. But also, and this is an important distinction we address here, for the magnitudes or sizes of many measurable objects or entities, such as firmament voids, lengths of rivers, city populations, etc. [6].

Here we analyze the conceptual, and also quantitative, difference between frequency and size ranked data. To this purpose we make use of a straightforward stochastic procedure [7–9] to reproduce ranked data from an assumed parent distribution that governs sets of values of random variables that constitute samples. Examination of the expressions for the two types of rank functions indicate that they are functional inverses of each other. See also [6, 10, 11]. In particular, we focus in the case where the parent distribution *P*(*N*), where *N* is a magnitude random variable, has the power-law form *P*(*N*) ∼ *N*^−*α*^, 1 ≤ *α* < ∞. We find that in the limit *α* = 1 the size-rank distribution *N*(*k*), where *k* is the rank, decays exponentially as *k* grows, while the frequency-rank distribution *F*(*k*′) decays logarithmically as *k*′, the corresponding rank variable, increases. On the contrary, in the limit *α* → ∞ *N*(*k*) decays logarithmically while *F*(*k*′) does so exponentially. The intermediate case *α* = 2 is the special exponent value when both *N*(*k*) and *F*(*k*′) decay as a power law with exponent −1, the classical Zipf’s power law value. To complement our description we replicate the procedure by considering instead a starting parent distribution *Q*(*F*) ∼ *F*^−*β*^, 1 ≤ *β* < ∞ where *F* is a frequency random variable, and obtain an equivalent account with 1 − *β* = 1/(1 − *α*).

**Fig 1:**
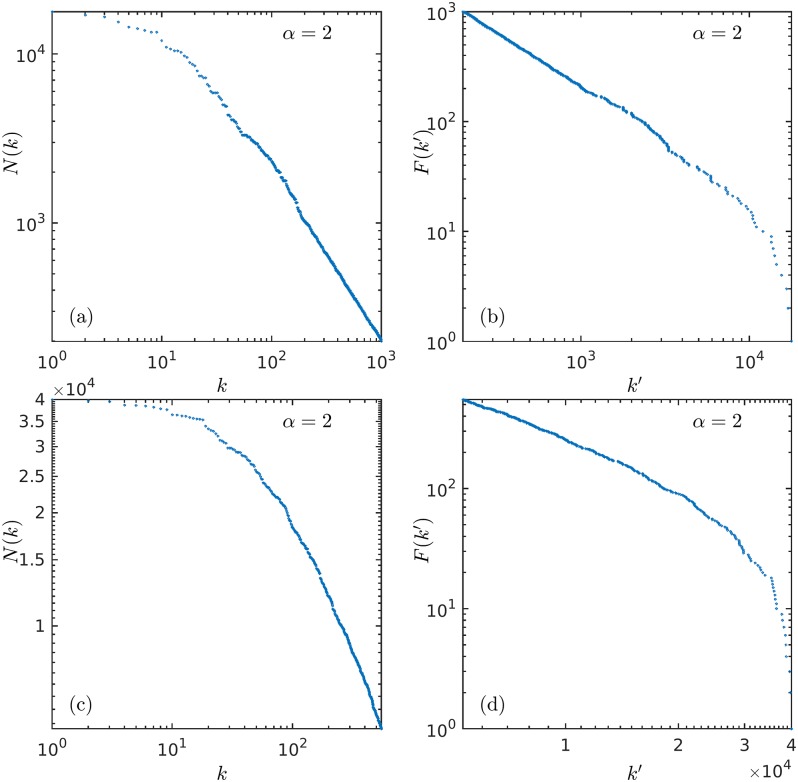
Two examples of ranked data. (a) Data for the energy released by earthquakes in California [14]. (b) Same earthquake data ranked according to number of occurrences of earthquakes of similar magnitude showing behavior compatible with the Guttenberg-Richter law. (c) Data for the areas burnt in forest fires in the U.S.A. [15]. (d) Same forest fires data ranked according to the number of occurrences of similar burnt areas. See text for description.

We have recently [8, 9, 12] shown that the above-referred stochastic approach to size-rank distributions can be exactly represented by deterministic nonlinear one-dimensional iterated maps close to tangency [13]. Here we extend this strict analogy to determine frequency-size distributions within this nonlinear dynamical language. These distributions are given by areas below map trajectories. To explore the duality between size-rank *N*(*k*) and frequency-rank *F*(*k*′) distributions, we look at specific sets of real data that can be sorted out in both ways, magnitudes or numbers of occurrences, such as the cases of earthquakes [14] and forest fires [15] (see Fig 1), and we find agreement with the theoretical approach. We also comment on how Benford’s law [16, 17] for the frequency of digits corresponds in our scheme to the case *α* = 1.

Finally, we extend our statistical-mechanical interpretation with generalized entropies of rank distributions [12, 18] to include the role of *F*(*k*′).

## Rank distributions from a size parent distribution

The basic ingredient in the stochastic method [7–9] for rank distributions is the probability distribution *P*(*N*) of the magnitude or size data *N* under consideration. The scheme is phenomenological since the form of *P*(*N*) is assumed, and so, the first common choices are: gaussian, exponential, or power law expressions. For the latter case we write

$$\begin{array}{c} P\left(N\right)∼N^{-\alpha },\;1\leq \alpha <\infty . \end{array}$$ (1)

Sets of data *N* can be generated from Eq (1) and subsequently examined if they match, statistically, real ranked data sets. Each data set formed by a total of N entries, expressed with given suitable precision, can be ranked according to their sizes *N* or the numbers of times *F* with which their items appear. We shall consider that *N* takes positive values within an interval *N*_min_ ≤ *N* ≤ *N*_max_, where we allow as limiting values *N*_min_ = 0 and/or *N*_max_ → ∞. To obtain the number of occurrences *F* for real numbers *N* recorded with a given precision it may be necessary to introduce a partition and count incidences within intervals.

The entries in the sample set N can be sorted out starting with the largest, *N*_max_, and continuing with decreasing magnitudes down to *N*_min_. And then labeled with the rank variable variable *k*, with *k* = 0 for *N*_max_ and *k* = *k*_max_ for *N*_min_. We call the function *N*(*k*) the size-rank distribution. The rank *k* can be an integer *k* = 0, 1, 2, 3, …, *k*_max_ (often, elsewhere, the 1st value is *k* = 1) and it can be generalized to be a real number. The set N can also be ordered in terms of the frequency with which they appear, that is, the number of occurrences *F* having size equal or greater than *N*, or equivalently the rate *f*, 0 ≤ *f* ≤ 1, of occurrences having size equal or greater than *N*. For this second sorting the occurrences are labeled with a rank variable *k*′, with *k*′ = 0 for the most frequent and $k^{\prime }=k_{\max}^{\prime }$ for the least frequent. We call *F*(*k*′) the frequency-rank distribution. Similarly, the rank *k*′ can be an integer $k^{\prime }=0,1,2,3,\ldots ,k_{\max}^{\prime }$ (often the 1st value is *k*′ = 1) but it can be generalized to be a real number. The normalized frequency-rank distribution is $f\left(k^{\prime }\right)=F\left(k^{\prime }\right)/N$. The main task is to determine *N*(*k*) and *F*(*k*′) from *P*(*N*).

We now introduce the complementary cumulative distribution of *P*(*N*),

$$\begin{array}{c} \Pi \left(N,N_{\max}\right)=\int_{N}^{N_{\max}}P\left(N^{\prime }\right)dN^{\prime }, \end{array}$$ (2)

where the normalization of *P*(*N*) implies Π(*N*_min_, *N*_max_) = 1. The parent distribution *P*(*N*) can be recuperated from Π(*N*, *N*_max_) via

$$\begin{array}{c} P\left(N\right)=-\frac{\partial }{\partial N}\Pi \left(N,N_{\max}\right). \end{array}$$ (3)

In the theoretical approach the evaluation of Π(*N*, *N*_max_) is the means by which the values *N* generated by *P*(*N*) are sorted out and leads to the rank distributions.

The cumulative distribution Π(*N*, *N*_max_) increases monotonically as *N* decreases, taking values from Π(*N*_max_, *N*_max_) = 0 to Π(*N*_min_, *N*_max_) = 1. This distribution Π(*N*(*k*), *N*_max_), where we have now indicated the rank *k* occupied by the variable magnitude *N*, is identified with k/N, that is

$$\begin{array}{c} \frac{k}{N}\equiv \Pi \left(N\left(k\right),N_{\max}\right). \end{array}$$ (4)

The size-rank distribution *N*(*k*) is obtained by solving

$$\begin{array}{c} \frac{k}{N}=\int_{N(k)}^{N_{\max}}P\left(N^{\prime }\right)dN^{\prime }, \end{array}$$ (5)

for *N*(*k*). Normalization of *P*(*N*) indicates that $k_{\max}=N$. If *k* is to be an integer the possible lower limits in the integral in Eq (5), *N*(1), *N*(2), …, *N*(*k*_max_) are such that the integral takes values 1/N, 2/N, …, $k_{\max}/N$.

On the other hand, the fraction k/N can also be seen as the rate or scaled frequency with which the sizes equal or greater than *N* occur, small for small *k* ≃ 0 and large for *k* ≃ *k*_max_. Therefore we identify the normalized frequency-rank distribution *f*(*k*′) as

$$\begin{array}{c} f\left(k^{\prime }\right)\equiv \Pi \left(N,N_{\max}\right), \end{array}$$ (6)

where *k*′ ≡ *N*. If *k*′ is to be an integer the values of *N* to be used in *P*(*N*) are integers. In practice, the non-normalized frequency-size distribution $F\left(k^{\prime }\right)\equiv Nf\left(k^{\prime }\right)$ is often used as it is constructed directly from the numbers of occurrences in data samples. From the above definitions *k*′ ≡ *N*, and $F\left(k^{\prime }\right)\equiv Nf\left(k^{\prime }\right)$, together with Eqs (4) and (6), it is clear that the rank distributions *N*(*k*) and *F*(*k*′) are functional inverses of each other. That is, *k* = *F*(*N*) or *N* = *F*^−1^(*k*). The inverse of a cumulative distribution is referred to as the quantile function [6, 10]. We refer to *N*(*k*) as the size-rank distribution even though technically it is not a probability distribution, as *P*(*N*) and *f*(*k*′) are.

## Rank distributions from a power-law parent distribution

We look now at the specific expressions that come out of the general equations in the previous Section when *P*(*N*) is given by Eq (1). We have

$$\begin{array}{ccc} \Pi (N\left(k\right),N_{\max}) & = & \int_{N(k)}^{N_{\max}}N^{-\alpha }dN\\ & = & \frac{1}{1-\alpha }\left[N_{\max}^{1-\alpha }-N{\left(k\right)}^{1-\alpha }\right], \end{array}$$ (7)

or, in terms of the *q*-deformed logarithmic function ln_*q*_(*x*) ≡ (1 − *q*)^−1^[*x*^1−*q*^ − 1] with *q* a real number,

$$\begin{array}{c} \ln_{\alpha }N\left(k\right)=\ln_{\alpha }N_{\max}-N^{-1}k. \end{array}$$ (8)

The size-rank distribution *N*(*k*) is explicitly obtained from the above with use of the inverse of ln_*q*_(*x*), the *q*-deformed exponential function exp_*q*_(*x*) ≡ [1 + (1− *q*)*x*]^1/(1 − *q*)^, this is

$$\begin{array}{c} N\left(k\right)=N_{\max}\exp_{\alpha }\left[-N_{\max}^{\alpha -1}N^{-1}k\right]. \end{array}$$ (9)

While the frequency-rank distribution *f*(*k*′) is given by

$$\begin{array}{ccc} f(k^{\prime }) & = & \ln_{\alpha }N_{\max}-\ln_{\alpha }k^{\prime }\\ & = & 1+\ln_{\alpha }N_{\min}-\ln_{\alpha }k^{\prime }. \end{array}$$ (10)

**Fig 2:**
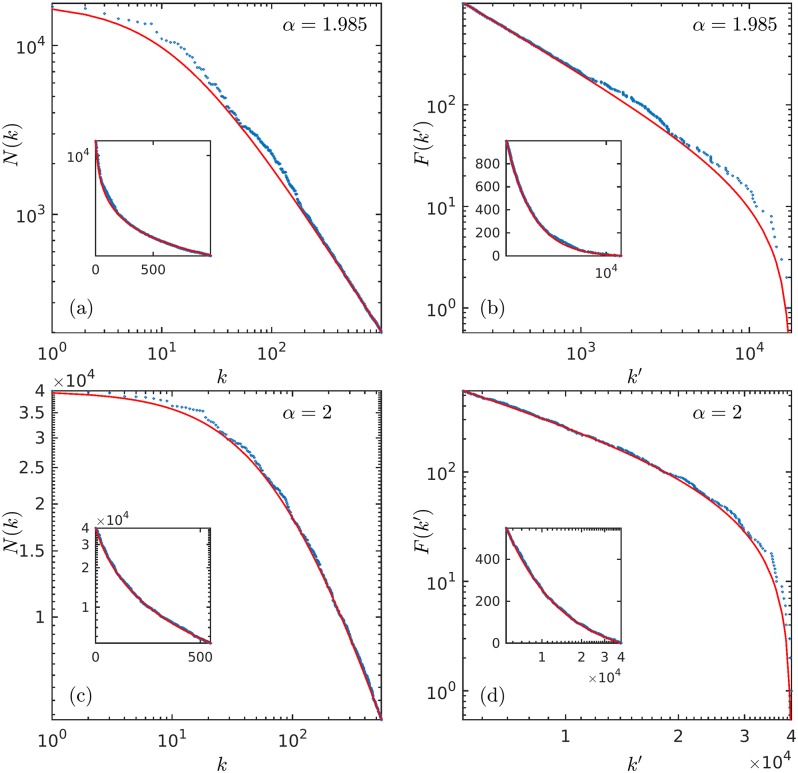
Theoretical fitting of ranked data. Same two examples in Fig 1 of ranked data on earthquakes and forest fires fitted with the expressions in Eqs (9) and (10). (a) Size-rank distribution *N*(*k*) for earthquakes. (b) Frequency-rank distribution for earthquakes (with $F\left(k^{\prime }\right)=Nf\left(k^{\prime }\right)$). (c) Size-rank distribution *N*(*k*) for forest fires. (d) Frequency-rank distribution for forest fires (with $F\left(k^{\prime }\right)=Nf\left(k^{\prime }\right)$). As can be seen, the values of *α* needed for fitting are close to *α* = 2 that corresponds to the classical Zipf law exponent. See text for description.

In Fig 2 we show the agreement of Eqs (9) and (10) with the data on earthquakes and forest fires already shown in Fig 1. Our method for fitting the data to Eqs (9) and (10) is heuristic. We first select a data point to define *N*_max_. We then approximate with a straight line segment a section of the data that appears lined when displayed in logarithmic scales (involving a choice of its two extremes) via minimum squares. This gives us, with the use of Eq (8), a set of two equations from which we determine numerically preliminary values for *α* and N (notice that Eq (7) has no normalization constant). We iterate this procedure to improve fitting (mostly only N changes its value appreciably). Once the parameters in Eq (9) are determined *F*(*k*′) follows from Eq (10).

When *α* = 1 Eq (9) acquires the ordinary exponential form

$$\begin{array}{c} N\left(k\right)=N_{\max}\exp\left(-N^{-1}k\right), \end{array}$$ (11)

while Eq (10) becomes an ordinary logarithmic function,

$$\begin{array}{ccc} f(k^{\prime }) & = & \ln\;(N_{\max}/k^{\prime })\\ & = & 1-\ln\;(k^{\prime }/N_{\min}). \end{array}$$ (12)

We take the limit *α* → ∞ to signify that *P*(*N*) = *N*_0_exp(−*N*_0_ *N*), and we choose *N*_0_ = 1. We find

$$\begin{array}{c} N\left(k\right)=-\ln[\exp\left(-N_{\max}\right)+N^{-1}k], \end{array}$$ (13)

and

$$\begin{array}{ccc} f(k^{\prime }) & = & \exp\left(-k^{\prime }\right)-\exp\left(-N_{\max}\right) \end{array}$$ (14)

$$\begin{array}{ccc} & = & \exp\left(-k^{\prime }\right)-\exp\left(-N_{\min}\right)+1. \end{array}$$ (15)

In the limit *N*_max_ → ∞ Eq (9) becomes the power law *N*(*k*) ∼ *k*^1/(1 − *α*)^ that when *α* = 2 gives the simple hyperbolic form *N*(*k*) ∼ *k*^−1^. Whereas Eq (10) in the same limit becomes the power law *f*(*k*′) ∼ *k*′^(1−*α*)^ that when *α* = 2 gives, coincidentally, the same hyperbolic form *f*(*k*′) ∼ *k*′^−1^. For many sets of frequency-rank real data *α* ≃ 2 and the standard Zipf law is *α* = 2, whereas the same feature for real size-rank data has led to refer (concurrently) to the observation of Zipf’s law in relation to *N*(*k*). In contrast, when *α* → ∞, in the limit *N*_max_ → ∞ the rank distributions become N(k)=ln(N/k) and *f*(*k*′) = exp(−*k*′), *N*(*k*) decays very fast as *k* increases since the argument in the logarithmic function lies in the interval 0<k/N<1, while *f*(*k*′) decays exponentially as *k*′ increases. This can be compared with the case *α* = 1, but *N*_max_ finite, when *N*(*k*) decays exponentially as *k* increases while *f*(*k*′) decays very fast as *k*′ increases since again the argument in the logarithmic function lies in the interval $0<k^{\prime }/N_{\max}<1$.

A note on normalization. The choice of *P*(*N*) given by Eq (1) is not compatible with finite data sets (N<∞), these should be represented by a different expression for *P*(*N*), at least one that differs from Eq (1) for some values of *N*, specially small *N*. Normalization of Eq (1) obeys $k_{\max}=N$, with both *k*_max_ → ∞ and N→∞, while *N*_min_ → 0.

## Rank distributions from a frequency parent distribution

To show a duality feature of the approach to rank distributions we now consider the derivation of these distributions from a different parent distribution. This distribution, *Q*(*F*), generates values of the numbers of occurrences *F* to form data sets. As before we introduce a complementary cumulative distribution

$$\begin{array}{c} X\left(F,F_{\max}\right)=\int_{F}^{F_{\max}}Q\left(F^{\prime }\right)dF^{\prime }, \end{array}$$ (16)

where the normalization of *Q*(*F*) implies *X*(*F*_min_, *F*_max_) = 1. We denote by F the total number of elements in the occurrences sample set.

Proceeding as before we indicate the rank *k*′ occupied by the number of occurrences *F* in the distribution *X*(*F*(*k*′), *F*_max_) and identify this as $k^{\prime }/F$. That is

$$\begin{array}{c} \frac{k^{\prime }}{F}\equiv X\left(F\left(k^{\prime }\right),F_{\max}\right). \end{array}$$ (17)

When we assume the power law expression

$$\begin{array}{c} Q\left(F\right)∼F^{-\beta },\;1\leq \beta <\infty , \end{array}$$ (18)

we obtain

$$\begin{array}{c} X\left(F\left(k^{\prime }\right),F_{\max}\right)=\frac{1}{1-\beta }\left[F_{\max}^{1-\beta }-F{\left(k^{\prime }\right)}^{1-\beta }\right], \end{array}$$ (19)

or, in terms of the *q*-deformed logarithmic function,

$$\begin{array}{c} \ln_{\beta }F\left(k^{\prime }\right)=\ln_{\beta }F_{\max}-F^{-1}k^{\prime }. \end{array}$$ (20)

The frequency-rank distribution *F*(*k*′) is explicitly obtained from the above with with use of the *q*-deformed exponential function, this is

$$\begin{array}{c} F\left(k^{\prime }\right)=F_{\max}\exp_{\beta }\left[-F_{\max}^{\beta -1}F^{-1}k^{\prime }\right]. \end{array}$$ (21)

While the size-rank distribution *N*(*k*), following arguments parallel to those given before for *F*(*k*′), is given by

$$\begin{array}{c} N\left(k\right)\equiv FX\left(k,F_{\max}\right), \end{array}$$ (22)

where *k* ≡ *F*. Explicitly,

$$\begin{array}{ccc} N(k) & = & \ln_{\beta }F_{\max}-\ln_{\beta }k\\ & = & 1+\ln_{\beta }F_{\min}-\ln_{\beta }k. \end{array}$$ (23)

Again, it is clear that the rank distributions *F*(*k*′) and *N*(*k*) are functional inverses of each other. That is, *k*′ = *N*(*F*) or *F* = *N*^−1^(*k*′).

The exponent *α* in the previous two sections and the exponent *β* in this section are related via

$$\begin{array}{c} 1-\alpha =\frac{1}{1-\beta }, \end{array}$$ (24)

and coincide in value when *α* = *β* = 2, and both distributions acquire the simple hyperbolic functions *F*(*k*′) ∼ *k*′^−1^ and *N*(*k*) ∼ *k*^−1^ when in addition *F*_max_ → ∞ and *N*_max_ → ∞, that is, the classical Zipf case.

## Rank distributions from a nonlinear map at tangency

We have shown recently [8, 9, 12] that there is an exact analogy between the expressions for the rank distributions as presented above for *N*(*k*) and those for the trajectories associated with the tangent bifurcation in one-dimensional nonlinear iterated maps. A map *g*(*x*) at the tangent bifurcation is written locally as *x*′ = *g*(*x*) = *x*−*u*|*x*|^*z*^ + ⋯, *x* ≤ 0 [13], *z* > 1, and trajectories initiated at $x_{0}≲0$ are obtained via repeated iterations of *g*(*x*), i.e.

$$\begin{array}{c} x_{\tau +1}=x_{\tau }-u{|x_{\tau }|}^{z},\tau =0,1,\ldots \end{array}$$ (25)

These trajectories move monotonically towards the point of tangency at *x* = 0. If we make the replacement, valid for large time *τ*, of the difference *x*_*τ*+1_ − *x*_*τ*_ by *dx*_*τ*_/*dτ* in Eq (25) (written as −*u*|*x*_*τ*_|^*z*^ = *x*_*τ*+1_ − *x*_*τ*_) we obtain the differential form *udτ* = −|*x*_*τ*_|^−*z*^ *dx*_*τ*_, and integration of both sides of it yields

$$\begin{array}{ccc} ut & = & \int_{x_{0}}^{x_{t}}\frac{dx_{\tau }}{-{|x_{\tau }|}^{z}}\\ & = & \frac{1}{1-z}\left[-{|x_{t}|}^{1-z}+{|x_{0}|}^{1-z}\right], \end{array}$$ (26)

or

$$\begin{array}{c} \ln_{z}|x_{t}|=\ln_{z}|x_{0}|-ut. \end{array}$$ (27)

The iteration number or time *t* dependence of all trajectories is obtained by solving the above for *x*_*t*_, i.e.

$$\begin{array}{c} x_{t}=x_{0}\exp_{z}\left[-{|x_{0}|}^{1-z}ut\right]. \end{array}$$ (28)

The equivalence of the trajectory positions *x*_*t*_ with the size-rank distribution *N*(*k*) is made clear by comparison of Eqs (27) and (28) with Eqs (8) and (9), respectively, together with the identifications *t* = *k*, $u=N^{-1}$, *x*_*t*_ = −*N*(*k*), *x*_0_ = −*N*_max_ and *z* = *α*. Also, comparison of the right-hand side of Eq (26) with that of Eq (7), taking into account Eq (6), indicates that the analog of the frequency-rank distribution *f*(*k*′) is the quantity

$$\begin{array}{ccc} A_{t} & = & \int_{x_{0}}^{x_{t}}\frac{dx_{\tau }}{-{|x_{\tau }|}^{z}}\\ & = & \ln_{z}|x_{0}|-\ln_{z}|x_{t}|, \end{array}$$ (29)

where −*x*_*t*_ plays the role of *k*′. In [8] it is pointed out that the trajectories given by Eq (28) have precisely the analytical form for all trajectories with generic *x*_0_ that are generated by the functional composition renormalization group fixed-point map [13, 19] at the tangent bifurcation. And therefore the areas *A*_*t*_ in Eq (29) have also the same property. That is, all trajectories of the fixed-point map for all *t* initiated at the generic position *x*_0_ obey Eq (28). Also Eq (29) enjoys the degree of universality given by the fixed-point map.

**Fig 3:**
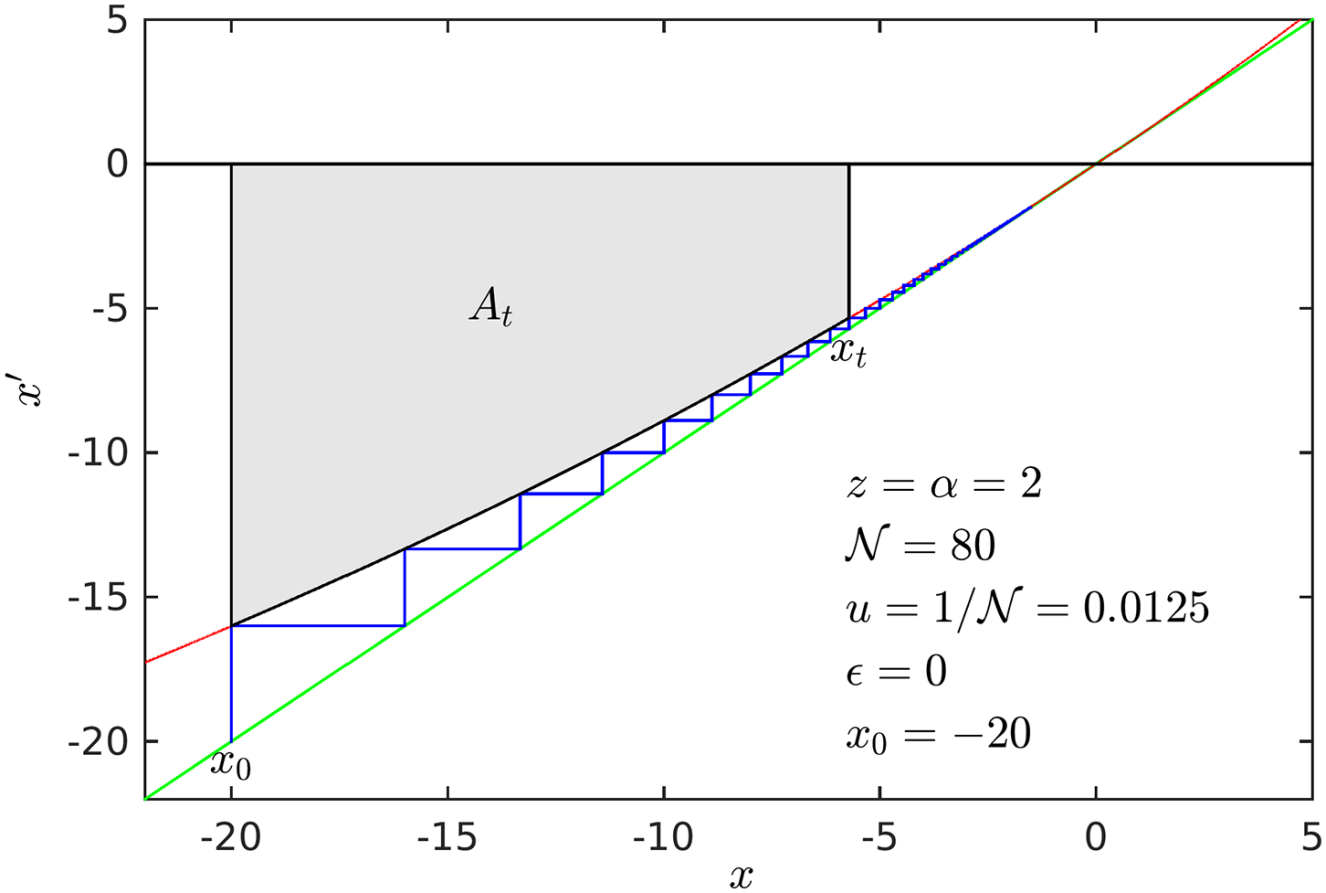
Iterated map at tangency. The map parameters are *z* = *α* = 2, the curvature is *u* = 0.0125, and the trajectory *x*_*t*_, *t* = 0, 1, 2, … *t*, is initiated at *x*_0_ as given by Eq (28). Also shown is the area *A*_*t*_ (shaded) as given by Eq (29). The map properties translate into the equivalent description of the rank distributions *N*(*k*) and *F*(*k*′) via the identifications *t* = *k*, $u=N^{-1}$, *x*_*t*_ = −*N*(*k*) = *k*′, *x*_0_ = −*N*_max_ and $A_{t}=F\left(k^{\prime }/N\right)$. See text for description.

In Fig 3 we illustrate the iterated map properties for the case *z* = *α* = 2 that translate into the equivalent description of the rank distributions *N*(*k*) and *F*(*k*′).

When *z* = 1 we have

$$\begin{array}{c} x_{t}=x_{0}\exp\left[-ut\right]. \end{array}$$ (30)

and

$$\begin{array}{c} A_{t}=\ln|x_{t}|-\ln|x_{0}|. \end{array}$$ (31)

The trajectories in Eq (30) are obtained when a linear map intersects the identity line, i.e.

$$\begin{array}{c} x^{\prime }=f\left(x\right)=\left(1-a\right)x, \end{array}$$ (32)

and this occurs locally when the tangent map is shifted into a double-secant map.

In the limit *z* → ∞ the counterpart of Eq (28) is

$$\begin{array}{c} x_{t}=\ln\left[\exp\left(x_{0}\right)+ut\right], \end{array}$$ (33)

as this expression transforms into Eq (13) for *N*(*k*) under the same equivalences *t* = *k*, $u=N^{-1}$, *x*_*t*_ = −*N*(*k*), *x*_0_ = −*N*_max_, while that corresponding to Eq (29) is

$$\begin{array}{c} A_{t}=\exp\left(x_{0}\right)-\exp\left(x_{t}\right). \end{array}$$ (34)

## Rank distributions associated with Benford’s first digit law

Benford’s first digit law [16, 17],

$$\begin{array}{c} \pi \left(n\right)=\log\left(\frac{n+1}{n}\right), \end{array}$$ (35)

where *n* is the first digit of a decimal base number *N* and log denotes the decimal base logarithmic function, can be readily expressed in terms of the complementary cumulative distribution Eq (2) when *α* = 1 and the parent distribution is *P*(*N*) = 1/*N*. This is

$$\begin{array}{c} \pi \left(n\right)=\Pi \left(N,N_{\max}\right)-\Pi \left(N+1,N_{\max}\right)=\log\left(n+1\right)-\log\left(n\right), \end{array}$$ (36)

where *N* + 1 = (*n* + 1).000⋯ and *N* = *n*.000⋯.

Thus, by considering the cumulative version of Benford’s law,

$$\begin{array}{c} \Pi \left(N,N_{\max}\right)=\log\left(N_{\max}\right)-\log\left(N\right), \end{array}$$ (37)

with *N*_max_ = 10 and *N* = *n*.000⋯, *n* = 1, 2, …, 9, we have

$$\begin{array}{c} F\left(k^{\prime }\right)=N\log\;\left(N_{\max}/k^{\prime }\right),k^{\prime }=1,2,\ldots ,N_{\max}, \end{array}$$ (38)

and

$$\begin{array}{c} N\left(k\right)=N_{\max}10^{-k/N},k=0,1,\ldots ,N_{\max}. \end{array}$$ (39)

**Fig 4:**
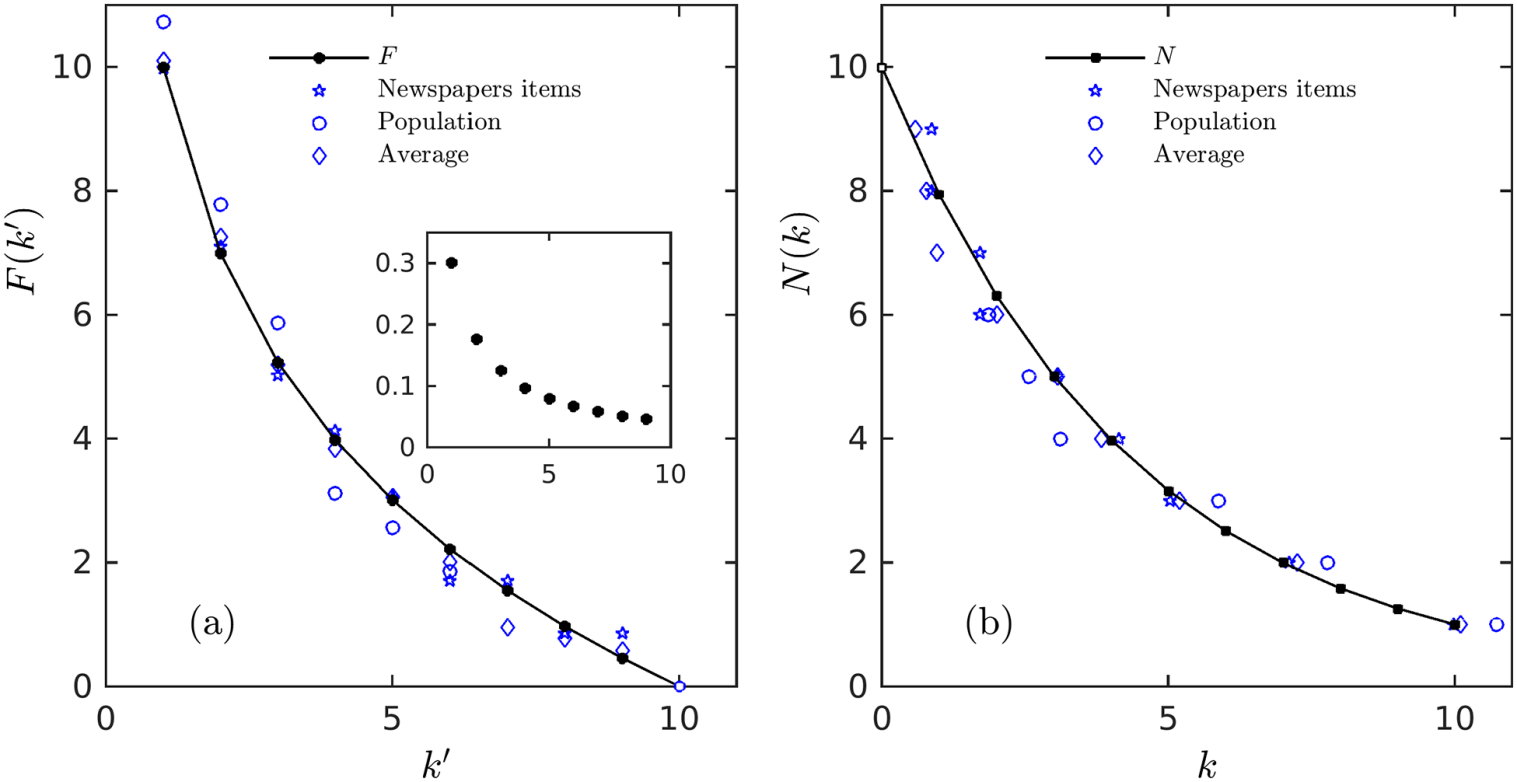
Rank distributions for Benford law. Rank distributions for Benford law together with numerical data that follows this law (shown in the inset). (a) Frequency-rank distribution *F*(*k*′). (b) Size-rank distribution *N*(*k*). They are obtained from the general formalism with *α* = 1. Data taken from Table I in the original Benford’s article Ref. [17]. See text for description.

In Fig 4 we show these distributions together with numerical data that follows Benford’s law as shown in the figure’s inset.

Benford’s law has been generalised to the case *α* > 1 [7], so that its associated (complementary) cumulative distribution Eq (7) provides the connection with the rank distributions studied here. In particular the case *α* = 2 corresponds to the classical Zipf’s law described by *F*(*k*′) with *k*′ = 0, 1, 2, 3, …, shifted and limited to the values of the first digits 1, 2, 3, …, 9, when using decimal base logarithms.

## Rank distributions as expressions of a thermodynamic structure

As pointed out *N*(*k*) is a the functional inverse of *F*(*k*′), that is, the inverse of a (non-normalized complementary) cumulative distribution in reverse order, a quantile function [6, 10]. Also *N*(*k*) has been interpreted [8, 9] as the total number that the size variable unit occurs at fixed rank *k*. That is, in thermal system language, *N*(*k*) is equivalent to the degeneracy of a micro state of ‘energy’ *k*, or a micro-canonical partition function with fixed *k*, where the associated uniform probability is $p_{i}^{\left(k\right)}=p^{\left(k\right)}\equiv 1/N\left(k\right)$ for all *i* = 1, …, *N*(*k*). Thus, we can call *S*(*k*) ≡ ln *N*(*k*) an entropy for *α* = 1 and define *S*(*k*) ≡ ln_*α*_ *N*(*k*) as a generalized entropy for *α* > 1. Likewise *S*_max_ ≡ ln *N*_max_ for *α* = 1 and *S*_max_ ≡ ln_*α*_ *N*_max_ for *α* > 1, *N*(0) ≡ *N*_max_. Eq (8) is written now as

$$\begin{array}{c} S_{\max}=S\left(k\right)+N^{-1}k, \end{array}$$ (40)

where, if *S*(*k*) is thought of as the entropy for the system with fixed *k*, then $S_{\max}\left(N^{-1}\right)$ would be a generalized Massieu potential when the variable *k* is replaced by the (conjugate) variable $N^{-1}$ via a Legendre transformation.

Just like thermodynamic quantities are dominant values of statistical-mechanical fluctuating quantities in a macroscopic system, we think of Eq (11), valid for *α* = 1,

$$\begin{array}{c} N_{\max}=N\left(k\right)\exp\left(N^{-1}k\right), \end{array}$$ (41)

to be the result of the application of the saddle-point approximation for large N on

$$\begin{array}{c} N_{\max}=\int N\left(k^{\prime }\right)\exp\left(N^{-1}k^{\prime }\right)dk^{\prime }. \end{array}$$ (42)

The consideration of the emergence of dominant rank fluctuations for the general case *α* > 1 in the ‘thermodynamic’ limit $k_{\max}=N\to \infty$ is less straightforward and here we do not discuss it further.

We recall [20, 21] that the formalism of thermodynamics can be expressed in two equivalent ways. One of them is to consider as starting point the entropy as the fundamental monotonic function of the energy (and other basic variables) that characterise the system, while the other alternative is to begin with the internal energy as the fundamental quantity, a monotonic function of the entropy (and the same other variables). The expressions obtained from the parent distribution *P*(*N*), Eqs (8) and (9), correspond to the former choice, while those obtained from the parent distribution *Q*(*F*), Eqs (20) and (21), relate to the second one.

In this statistical-mechanical interpretation the rank *k* plays the role of energy and the entropy is *S*(*k*) = ln *N*(*k*), when *α* = 1. In the alternative description *F* plays the role of energy while the entropy is ln(*k*′), again when *α* = 1. Thus here the quantities representing entropy and energy are, as customary, functional inverses of each other in accordance with the usual two equivalent thermodynamic frames [20, 21].

As a consequence of the precise analogy between the rank distributions obtained from a parent distribution and the nonlinear iterated fixed-point map at tangency, we note that the thermodynamic structure observed above for the rank distributions quantities translates thoroughly into an equivalent structure for the nonlinear dynamical problem. It is only necessary to recall the identifications *z* = *α*, *t* = *k*, $u=N^{-1}$, *x*_*t*_ = −*N*(*k*), *x*_0_ = −*N*_max_ and *A*_*t*_ = −*f*(*k*′) with *x*_*t*_ = −*k*′.

## Summary and discussion

We have analyzed the relationship that exists between two types of ranked data, numbers of occurrences and sizes or magnitudes of items. The technical relationship is well understood for statistics specialists, frequency-rank data is represented by a (complementary) cumulative probability distribution while size-rank data is described by its functional inverse, a quantile function [6, 10]. It is of wider interest, for those studying the many topics of the complex systems science, where universal patterns are observed in ranked data samples from very different sources, such as the empirical laws of Zipf and Benford, to understand the physical origin of the documented behavior We have obtained expressions for size-rank *N*(*k*) and frequency-rank *F*(*k*′) distributions from a stochastic method and or from an equivalent nonlinear deterministic approach and corroborated that the two functions are inverses of each other. Their differences are most apparent when the exponent *α* of the power-law parent distribution differs from *α* = 2, but they coincide and behave as hyperbolic functions (with deviations for small and large rank) when *α* = 2. In this latter case we have a nonlinear map at tangency with nonzero curvature, the most common case of analytic map at tangency. This being the case of the classic Zipf law. On the other hand we illustrated the case when *α* = 1 with the first digit Benford law.

We complemented our description by also considering the option for the parent distribution for the source of data to be that for the number of occurrences *F* instead of that for the size *N*. When these two distributions are assumed to have the power-law forms *P*(*N*) ∼ *N*^−*α*^ and *Q*(*F*) ∼ *F*^−*β*^ we obtain parallel (and equivalent) descriptions for the size and frequency rank distributions with the roles of cumulative distribution and quantile function interchanged and with the exponents relationship 1 − *α* = (1 − *β*)^−1^. Further, we advanced a thermodynamic and statistical-mechanical interpretation to be associated with the properties obtained for the rank distributions and indicated that the rank *k* plays the role of energy and *N*(*k*) takes the place in a prototypical thermal system of the number of configurations at fixed energy *k* with entropy *S*(*k*) = ln *N*(*k*) when *α* = 1. The interpretation of the alternative description corresponds to *F* playing the role of energy while ln(*k*′) that of entropy when *α* = 1. Thus entropy and energy as functional inverses of each other provide two equivalent thermodynamic formalisms [20, 21].

The case *α* > 1 suggests the use of the generalized entropy expression *S*(*k*) = ln_*α*_(*k*) in the thermodynamic description, but this poses a question for its corresponding statistical-mechanical formalism in that the validity of the usual saddle-point approximation requires reconsideration. The outcome may be one in which fluctuations are not suppressed in the thermodynamic limit, here represented by *k*_max_ → ∞ and N→∞. The reproduction of the rank distributions via a nonlinear map at a tangent bifurcation indicates a reason for the appearance of generalized entropy expression through the drastic contraction of configuration space from a real number set of possible iterated map trajectories positions to only a finite number in the limit *k*_max_ → ∞ and N→∞ that corresponds to *t* → ∞ [12, 18].
